# Decision Model for Allocation of Intensive Care Unit Beds for Suspected COVID-19 Patients under Scarce Resources

**DOI:** 10.1155/2021/8853787

**Published:** 2021-01-27

**Authors:** Eduarda Asfora Frej, Lucia Reis Peixoto Roselli, Rodrigo José Pires Ferreira, Alexandre Ramalho Alberti, Adiel Teixeira de Almeida

**Affiliations:** Universidade Federal de Pernambuco, Av. Acadêmico Hélio Ramos, s/n-Cidade Universitária, Recife, PE CEP 50740-530, Brazil

## Abstract

This paper puts forward a decision model for allocation of intensive care unit (ICU) beds under scarce resources in healthcare systems during the COVID-19 pandemic. The model is built upon a portfolio selection approach under the concepts of the Utility Theory. A binary integer optimization model is developed in order to find the best allocation for ICU beds, considering candidate patients with suspected/confirmed COVID-19. Experts' subjective knowledge and prior probabilities are considered to estimate the input data for the proposed model, considering the particular aspects of the decision problem. Since the chances of survival of patients in several scenarios may not be precisely defined due to the inherent subjectivity of such kinds of information, the proposed model works based on imprecise information provided by users. A Monte-Carlo simulation is performed to build a recommendation, and a robustness index is computed for each alternative according to its performance as evidenced by the results of the simulation.

## 1. Introduction

The coronavirus disease 2019 (COVID-19) has been affecting the whole world and changing the routines of society in many cities. The COVID-19 disease, caused by coronavirus (SARS-CoV-2), is very contagious and has spread rapidly in many cities around the world. This disease is exhibited in different forms in the human organism and, in severe cases, results in an acute respiratory syndrome, which requires treatment in hospital Intensive Care Units (ICUs), with the support of specific equipment, such as mechanical ventilation [1–3]. The rapid spread of COVID-19 disease leads to a chaotic scenario, whereby an imbalance arises between the number of severe cases requiring treatment and the limited number of resources available to treat these patients.

In this context, the COVID-19 pandemic brings critical medical decisions related to the restriction of resources available in healthcare systems around the world, and doctors take responsibility for such decisions when deciding how to best allocate those scarce resources. Hence, the proposition of structured decision-making models based on well-founded elements of decision theory may be of great interest for health systems managers during the COVID-19 pandemic.

Therefore, this study is aimed at presenting a decision model to support an important medical decision faced daily by doctors in health system routines during the COVID-19 pandemic, considering that scarce hospital resources are available: the ICU beds allocation problem. Doctors usually make decisions based on their personal judgments and previous experience; therefore, the proposed approach is aimed at getting this knowledge and previous experiences as input for a decision model, which seeks to provide a structured and rational framework for the decision-making process with regard to critical decision problems that deal with human lives.

Decision analysis techniques have been widely used to support how to structure and resolve medical decision-making problems [4–8]. For example, Jiang et al. [6] and Xu et al. [8] use the decision tree technique to support how best to structure the screening problem. In the same context, Janssen et al. [9] perform a literature review on methods which were used to structure the screening problem. Roselli et al. [10] tackled the screening problem for patients with suspected/confirmed COVID-19, considering a multiattribute model based on the utility theory.

Therefore, in this study, decision analysis concepts [11–14] are used to construct a utility-based model for handling the ICU bed allocation problem. A risk scenario is considered, which is related to the prognosis as to whether the patient is likely to survive or die. Also, subjected probabilities are considered [14], estimated by doctors in the form of imprecise information, the doctors being the decision-makers in these decision-problems. Due to the inherent imprecision of the information about chances of survival in different scenarios, the proposed model works based on the Monte-Carlo simulation in order to calculate a robustness index for each alternative. Therefore, the model provides doctors with recommendations regarding the medical decision problem investigated. The proposed approach is operationalized by a decision system which is freely available for use of doctors in health systems all over the world, and a database was designed to store the data of every occurrence, which will be analyzed and investigated in future research.

The main contribution of the present work relies on giving focus to the scarce resources scenario faced by health systems all over the world during the COVID-19 pandemic, which is overcharging health units and forcing doctors to make hard decisions on how to allocate those scarce resources. A specific modeling approach based on portfolio selection tools specifically directed for the COVID-19 context is developed in this paper, different from previous generic approaches present in the literature (e.g., Almeida et al. [15]). Moreover, a web-based decision information system was developed as the main product of this work, which can be easily used by health professionals all over the world.

This paper is organized as follows. “The ICU Allocation Problem” presents a brief review on the Intensive Care Units allocation problem and its relevance, highlighting its important aspects. “Portfolio-Based Approach for the ICU Allocation Problem” presents the mathematical model proposed to address the ICU allocation problem, which is based on a portfolio selection approach. “Allocation of ICU Beds under Scarce Resources: Practical Application” describes how the proposed model is operationalized, as well as a practical application of the proposed approach. Finally, “Final Remarks” presents the conclusions and final remarks of this work.

## 2. The ICU Allocation Problem

The ICU allocation problem has been investigated in the literature for a long time, since the resources for critical care are limited and cost-intensive. It is not uncommon to experience a situation where the number of ICU beds available is less than the number required to attend to patients who require them: the availability of this scarce resource is highly impacted by stochastic patient demands and stochastic service times, in a way that makes managing such a resource a complex problem [16, 17].

In this context, a model that aims at maximizing the expected number of lives of patients in a Pediatric ICU has been preliminary proposed by Almeida et al. [15]. Giannini and Consonni [18] investigated the perceptions and attitudes of health professionals regarding inappropriate admissions and resource allocation in the intensive care setting. They conducted a survey by applying a questionnaire to ICU doctors in Milan, Italy, and observed that 86% of them recognized that there were inappropriate admissions to ICU beds. This was due to several reasons, such as clinical doubt, limited time to make decision, errors in assessment, and pressure from superiors. Also, 5% of the respondents reported refusing appropriate admission due to financial issues. Finally, 67% reported that they frequently received requests to admit patients to ICU installations when no beds were available.

Concerned about the risk of occupational stress, Coomber et al. [19] performed a study to investigate the occupational stress of ICU doctors in the United Kingdom: they conducted a postal survey and observed that 29% of the respondents could be considered distressed, while 12% of them could be considered depressed. Thus, supporting doctors to deal with the ICU allocation problem by conducting this decision process in an easy, structured and rational way, may be very opportune, since this decision process is not trivial and is undertaken in a stressful situation.

Therefore, some studies presented in the literature propose techniques or models to support the ICU allocation problem. He et al. [20] presented a systematic literature review of research design and modeling techniques to support inpatient bed management. The authors recognized the complexity of this problem, which is affected by several factors, such as uncertainties about the patients' length of stay, fluctuations in demands, and unexpected admissions. They verified that simulation has been the main tool used in studies in this area. Reiz et al. [21] discussed the use of big data and machine learning to improve the way the ICU allocation problem is handled.

Shmueli and Sprung [22] investigated the survival benefits of allocating a patient to an ICU. The authors pointed out that in a situation of resource limitation, the policies for ICU admission should distinguish between the probability of survival and the benefits of survival. In their field study, which was undertaken in the ICU of the Hadassah-Hebrew University Medical Center during a seven-month period, the benefit was computed by using a model which considers admission and survival variables combined with the observable characteristics of patients. As a result of this study, the authors concluded that the ICU admission policy practiced until that moment in that hospital does not maximize the potential survival benefits.

On the other hand, Edenharter et al. [17] pointed out that most of the literature about the ICU allocation problem deals with the admission problem, but few studies tackle supporting the discharge decision problem. They investigated the ICU discharge problem: a univariate logistic regression model was proposed in order to assess the impact of the length of stay in the ICU, using data from two surgical ICUs of a large academic medical center. They observed that the absence of appropriate beds in the regular ward is the main cause of the delay in ICU discharge. They emphasized that this problem is of economic and ethical relevance, since the resources of the ICU are scarce. Azcarate et al. [23] also focused on the ICU discharge problem: they present a review of the literature on patient discharge decisions and propose a simulation framework that enables the real-world processes for discharging patients to be modeled in a more realistic way.

Some studies have addressed the ethical issues inherent to the ICU allocation problem: Oerlemans et al. [24] conducted interviews with health professionals concerning ethical problems, such as how full ICU occupancy and treatment decisions are reached in terms of choosing what patients should benefit from them. Health professionals' attitudes were collected in order to provide insights to improve the management of intensive care resources. As a conclusion, the authors suggested that the collective responsibility and effort by health professionals (ICU professionals and different professionals in the wards) have to be reinforced in a hospital routine in order to alleviate moral distress caused by the ethical dilemmas faced, since these two factors are mutually dependent on each other. Consequently, health professionals have to work together for an optimal transfer of patients between hospital departments. McGuire and McConnell [25] also discuss fairness and ethics in the ICU allocation problem, suggesting that an alliance of ethical and moral principles has to be applied in order to obtain a moral, ethical, and common-sense approach to deal with this complex problem.

The ICU allocation problem acquires a special dimension when there are public health emergencies, which can be caused by several factors, such as natural disasters and major outbreaks of infectious diseases [26, 27]. Christian et al. [28], concerned about outbreaks of avian influenza (H5N1), highlighted the importance of preparing a plan for allocating resources, such as mechanical ventilators, which can become scarce during a pandemic. The authors proposed a triage protocol for allocating resources for critical care during an influenza pandemic: the protocol uses the SOFA score and has four main components: inclusion criteria, exclusion criteria, minimum qualifications for survival, and a prioritization tool. The prioritization tool they proposed determines that the highest priority for accessing ICU beds be given to patients who meet the inclusion criteria and whose probabilities of survival are greatest.

Cao and Huang [27] created a discrete event simulation model to evaluate the performance of four principles that have been often proposed as alternatives to guide the allocation of scarce resources during a public health emergency. The four principles are as follows: First Come-First Served (FCFS), which recommends the allocation of the resources to the earliest arrivals; Random Selection (RAN), which recommends the random allocation of the resources; Most Serious First (MSF), which recommends the allocation of the resources to the most seriously ill patients; and Least Serious First (LSF), which recommends the allocation of the resources to the least seriously ill patients. The authors observed that the MSF principle is intuitively favored by many authors. However, according to the results that they obtained, among the four principles evaluated, this principle performs poorest, resulting in a greater death toll. On the other hand, the LSF principle presented the best performance based on the death toll in different scarcity scenarios. However, as the authors pointed out, this principle may be problematic from an ethical perspective.

The ethical issues involved in allocating resources during a public health emergency were analyzed by Ghanbari et al. [29] in a systematic literature review. The authors observed that several clinical and nonclinical factors have been considered in protocols to prioritize patients. However, there is no clear definition about the most appropriate principles that should underpin such a prioritization. Despite this lack, the authors highlighted the importance of maintaining clear and explicit guidelines for prioritizing limited resources, in order to improve how the general public perceives the basis for such prioritization.

This review demonstrates that the ICU allocation problem is not a trivial decision problem, first, because the scarcity of resources for intensive care cannot be overcome quickly because the cost of doing so is very high and there are shortages of appropriately qualified and experienced personnel and, secondly, because the nonallocation of a place in an ICU in some cases is likely to increase the probability that the patient will die. This problem involves ethical and financial issues for which there are no instantly applicable solutions, and this dilemma becomes all the more acute during public health emergencies in which mortality rates are expected to be much higher than at other times. The current example of this is the COVID-19 pandemic which is being experienced in several regions worldwide including in countries which simply do not have the budgetary and human resources needed for intensive care. This situation leads to health professionals involved in patient care working under very stressful conditions, which can hinder rational decision-making in line with policies established by public health authorities. This paper presents important contributions for this context: a decision model based on the utilitarian principle [29] is presented for the ICU allocation problem. The approach that we propose seeks to save the largest possible number of lives, by maximizing the expected number of lives saved in all groups analyzed by defining guidelines on to which patients ICU beds should be allocated. In order to support the use of the proposed model, a system that is available online for free is presented as a tool that aids operationalizing the proposed methodology. In summary, Table 1 presents an overview to support the understanding of previous approaches related to ICU allocation issues.

## 3. Portfolio-Based Approach for the ICU Allocation Problem

The ICU allocation model addresses the following situation: there are *n* candidate patients for occupying *w* available ICU beds (in which *w* < *n*), and the doctor should decide which of these patients are going to be allocated into ICU beds, considering their chances of survival in the ICU and outside the ICU.

Before introducing the mathematical model, an important issue that should be highlighted here is how the input data is given by the users. The input given by the doctors concerns the chances of survival of the patient in different scenarios: the user estimates the chances of survival of every candidate patient in the ICU and outside the ICU.

It is not trivial, however, for the user to provide these probability estimations. It may be hard for a doctor, even after analyzing the patient's clinical state and symptoms, to establish probabilities of survival in these different scenarios. This information is extremely subjective and may be imprecise. This type of medical decisions inherently involve uncertainty into the model. Such uncertainties sometimes derive from a random pattern of the variable being analyzed, as well as lack of knowledge and/or lack of understanding about a future condition. De Almeida et al. [14] list some factors from which uncertainties may arise, such as inaccuracy of measurement techniques, lack of details, and lack of data, among others. For the medical decision problem treated in this paper, there is uncertainty related to what will happen to a patient's life, depending on the treatment conduction adopted with him/her. Therefore, probabilities of survival and death of a patient in certain treatment conditions should be estimated.

In decision theory, a key element of many decision problems is the prior probability of the state of nature (*θ*), and the so-called prior probability distribution (*π*(*θ*)) is shown as a convenient manner to quantify this information [30]. In the context of our decision-making problem, two states of nature are possible: patient survives or patient does not survive. Considering that two possible alternatives are available for such patient (allocate or not allocate an ICU bed for that patient), the prior probabilities for this problem can be represented as in Table 2.

The role of experts' knowledge in this process is crucial, since their experience about the variables of the decision problems can be used to estimate those prior probabilities [31]. According to Garcez et al. [32], a purely frequentist notion of probability cannot be applied in some cases, because some events are very rare, and therefore, their repetition is difficult to be predicted, especially when historical data is insufficient. Hence, it becomes impractical to estimate frequentist probabilities in such cases. According to Berger [30], subjective probabilities are not correct or accurate probabilities, but a measure of the degree of beliefs of the experts about the chance of occurrence of a particular event.

Clemen and Winkler [33] state that factors that have influence on the probabilities should be correlated with technical characteristics of the analyzed system. In order to do so, all the experience acquired by experts should by applied, considering their knowledge and expertise regarding the system. In this way, experts are able to provide valuable and insightful information for the decision problem being treated.

Experts' prior knowledge should, therefore, be elicited in order to be useful for the decision problem. Kadane and Wolfson [34] claim that the main purpose of the elicitation is to gather the main characteristics of the opinion of these experts and therefore to integrate their academic knowledge and previous experiences. Frequentist inference allows the interpretation of probabilities, while the Bayesian approach for statistics is completely based on subjective or personal interpretations of probabilities [35].

Therefore, the proposed approach considers probabilities of survival of a patient inside and outside ICU as prior probabilities, which are estimated by doctors, who act as experts in this case, considering their prior knowledge and experience about the situation. Hence, our model assumes that the doctor will be able to specify a measure the probability of survival of each patient *i* in the ICU (*π*_*i*_(*S*_IN_)) and outside the ICU (*π*_*i*_(*S*_OUT_)).

However, we recognize that such information inherently involves imprecision on its estimation. Therefore, our approach considers ranges of probabilities, instead of exact values of probabilities. Before designing the form of these input data, three doctors acted as specialists for this research and gave their opinion regarding the way in which they feel more comfortable and self-confident about providing such information. All of them stated that expressing these chances in a verbal scale makes them much more secure and comfortable than providing numbers does.

The combination of multiple experts' knowledge has advantages that were listed by Winkler et al. [36]. First, combined probability distributions leads to a better result than a single probability distribution (“two heads are better than one,” according to the psychological point of view). Second, the final probability distribution may be considered a way of agreement between different experts' knowledge. Finally, the analysis becomes more complete when several opinions are considered.

In this sense, a 5-point Likert scale (*very low*, *low*, *medium*, *high*, and *very high*) was built for users to estimate chances of survival in each specific scenario, as a consensus reached by those three doctors. Each level of this scale is then converted into a range of probabilities of survival. The option *very low* means the chance of survival varies from 0 to 20%; the option *low* covers from 20% to 40%; a *medium* chance is from 40% to 60%; a *high* chance of survival covers from 60% to 80%; and *very high* means that the patient survives with 80% to 100% probability. Within those ranges, a Monte-Carlo simulation is conducted for generating a recommendation for the user, based on a robustness index of each alternative, which is detailed later on in this paper.

As previously mentioned, the input data of this model are the probability of survival of each patient *i* in the ICU (*π*_*i*_(*S*_IN_)) and outside the ICU (*π*_*i*_(*S*_OUT_)). The users estimate these chances of survival using a verbal scale, and probabilities are given in ranges of 20% (quintiles) that are derived from levels of a 5-point Likert scale. The probabilities of death in and outside the ICU can be obtained by one minus the respective probability of survival.

The utility of survival in the ICU (*U*(*S*_IN_)) and outside the ICU (*U*(*S*_OUT_)) are parameters of the model which are considered the same for every patient, since the lives of all of them have the same value for the doctor/user. The utilities of death in the ICU *U*(*D*_IN_) and outside the ICU *U*(*D*_OUT_) are also parameters considered the same for every patient.

Let *X*_*i*_(*i* = 1, ⋯, *n*) be a binary decision variable, which indicates whether patient *i* goes to the ICU (*X*_*i*_ = 1) or patient *i* does not go to the ICU (*X*_*i*_ = 0). Then, the expected utility of patient *i* when he/she goes to ICU (*U*_*i*_(*X*_*i*_ = 1)) can be calculated as per Equation (1), and the expected utility of patient I when he/she does not go to ICU *U*_*i*_(*X*_*i*_ = 0) can be calculated using Equation (2).

(1) $$\begin{array}{c} U_{i}\left(X_{i}=1\right)=\pi_{i}\left(S_{\text{IN}}\right)\;x\;U\left(S_{\text{IN}}\right)+\pi_{i}\left(D_{\text{IN}}\right)\;x\;U\left(D_{\text{IN}}\right), \end{array}$$

(2) $$\begin{array}{c} U_{i}\left(X_{i}=0\right)=\pi_{i}\left(S_{\text{OUT}}\right)\;x\;U\left(S_{\text{OUT}}\right)+\pi_{i}\left(D_{\text{OUT}}\right)\;x\;U\left(D_{\text{OUT}}\right). \end{array}$$

In (1) and (2), the probabilities of death in ICU and outside ICU are calculated according to (3) and (4), respectively.

(3) $$\begin{array}{c} \pi_{i}\left(D_{\text{IN}}\right)=1-\pi_{i}\left(S_{\text{IN}}\right), \end{array}$$

(4) $$\begin{array}{c} \pi_{i}\left(D_{\text{OUT}}\right)=1-\pi_{i}\left(S_{\text{OUT}}\right). \end{array}$$

Given these utilities of staying inside and outside the ICU for each patient, the overall utility of the ICU allocation (*U*_ICU_(*X*_1_, *X*_2_, ⋯, *X*_*n*_)) can be calculated based on Equation (5), by the sum of the expected utility of each patient.

(5) $$\begin{array}{c} U_{\text{ICU}}\left(X_{1},X_{2},\cdots ,X_{n}\right)=\left[\overset{n}{\underset{i=1}{\sum }}U_{i}\left(X_{i}=1\right)\;x\;X_{i}\right]+\left[\overset{n}{\underset{i=1}{\sum }}U_{i}\left(X_{i}=0\right)\;x\;\left(1-X_{i}\right)\right]. \end{array}$$

In order to find the combination of patients that maximize the overall utility of the ICU allocation (*U*_ICU_(*X*_1_, *X*_2_, ⋯, *X*_*n*_)), a binary integer linear optimization model is run ((6)–(8)). The constraints of the optimization model are Equations (7) and (8). Equation (7) guarantees that the sum of patients that go to the ICU does not exceed the number of ICU beds (*w*), since Equation (8) imposes that the decision variables are binary.

(6) $$\begin{array}{c} Ma\text{x }U_{\text{ICU}}\left(X_{1},X_{2},\cdots ,X_{n}\right), \end{array}$$

s.t.

(7) $$\begin{array}{c} \overset{n}{\underset{i=1}{\sum }}X_{i}=w, \end{array}$$

(8) $$\begin{array}{c} X_{i}\in \left\{0,1\right\}\forall i=1,\cdots ,n. \end{array}$$

This optimization model recalls a portfolio selection model, and the output of this is the optimal combination of patients that should go to the ICU.

The design rationale for our bed allocation approach relies mainly on maximizing the number of lives saved, considering a scenario in which the health system is overcharged due to the effects of the COVID-19 pandemic. In order to do so, we consider a portfolio selection approach that takes into account expected utility concepts. The objective is to maximize the overall utility of the system, considering subjective probabilities of survival of each patient inside ICU and outside ICU. In order to model such situation, the survival scenario is considered the best situation, and therefore, the utility of survival in the ICU and outside the ICU is considered to have the maximum value of utility within the considered scale. In an analogous manner, the utility of death inside the ICU and outside the ICU is considered to have the lowest utility value within the considered scale. Our approach is based on the utilitarian principle that treats the lives of all patients with equal importance, without distinction between them. The following section describes how the proposed model is operationalized to be applied in practical cases.

## 4. Allocation of ICU Beds under Scarce Resources: Practical Application

The model presented in “Portfolio-Based Approach for the ICU Allocation Problem” is operated by means of a Decision Information System, which is freely available for users at http://insid.org.br/sidtriagem/app/. The software was developed in a web-based environment, with a user-friendly interface that allows doctors to interact with the platform. The software has mainly two operation modules: the computational module and the interactive module. The computation module of the software works according the calculations detailed in “Portfolio-Based Approach for the ICU Allocation Problem,” based on a Monte-Carlo simulation model. The interaction module works as explained in the following paragraphs. The system also has connection with a database, and the data of all occurrences performed are stored on it, in order to allow data analysis for future research to be performed.

It should be highlighted here that the role of the system is to act as a support tool for the users, but the final decision is always the responsibility of the user, who can choose whether or not to follow the recommendation given by the system.

**Figure 1:**
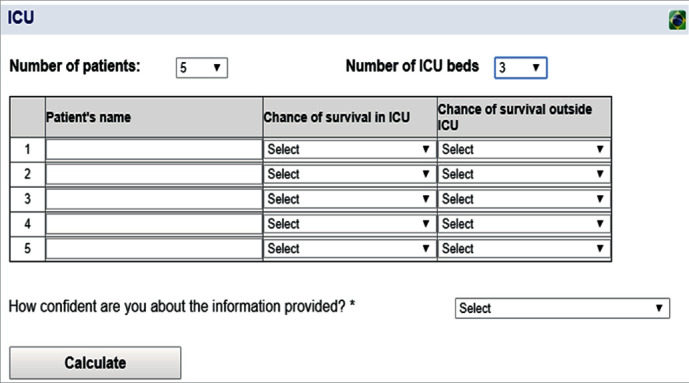
Input data for the ICU allocation problem.

Figure 1 shows the initial interface of the system. First, the user should enter the total number of candidate patients to go to the ICU (*n*) and the number of ICU beds available (*w*). Optionally, the user may enter the name of the patients. Then, the user should enter, for each patient, the chances of survival in the ICU and outside the ICU. The options given for the user are based on a 5-point Likert scale (*very low*, *low*, *medium*, *high*, and *very high*), which is converted into probability ranges of 20% (quintiles), as previously explained. Optionally, the user can also register how confident he/she feels in giving such information (*very unconfident*, *unconfident*, *neutral*, *unconfident*, *very unconfident*, or even *not applicable* (*N/A*)).

As previously mentioned, chances of survival in the ICU and outside the ICU are given by the user considering a 5-point Likert verbal scale, and the levels of the scale are converted into probability ranges (quintiles), as previously explained in “The ICU Allocation Problem.” Therefore, a Monte-Carlo simulation is performed in order to obtain a recommendation for the user. At each simulation instance, random values for probabilities are generated according to a uniform distribution within the respective range given by the input provided by the user. Then, the expected utilities are calculated for each patient and the optimization model in ((6)–(8)) is run to search for the optimal allocation of patients. At the end of the simulations, a robustness index is calculated for each patient, based on the number of simulation scenarios he/she appears in the optimal portfolio. The next topic shows a practical example to illustrate how the ICU module works.

**Figure 2:**
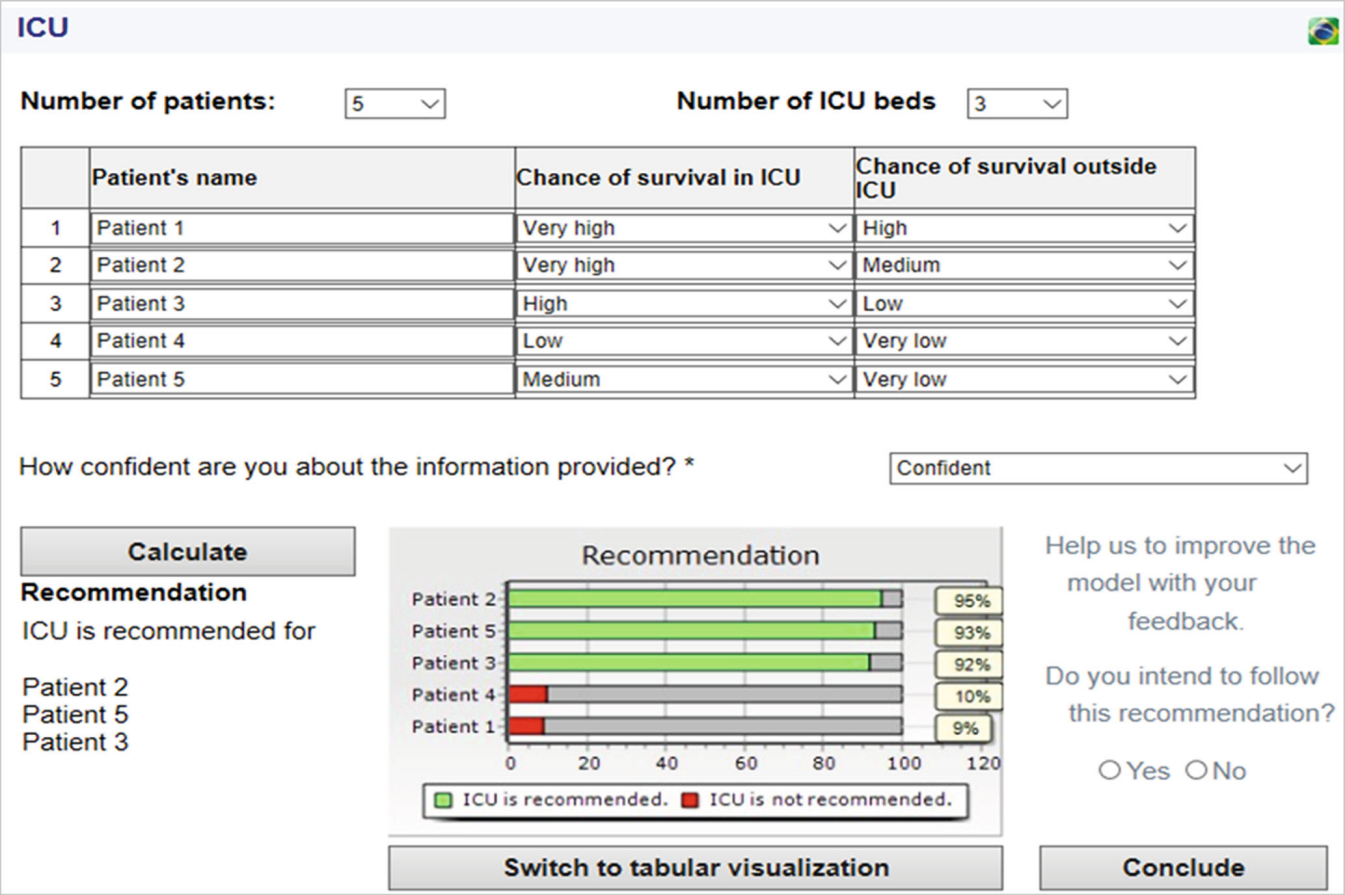
Practical example of the ICU allocation problem.

In order to illustrate the applicability of the proposed model, let us consider a hypothetical example in which there are 5 candidate patients for only 3 available ICU beds. The user should first enter the chances of survival in the ICU and outside the ICU for each of these patients, based on the symptoms, exams, and clinical assessment of each of them. Hypothetically, let us assume that “Patient 1” has a *very high* (80%-100%) chance of survival in the ICU and a *high* (60%-80%) chance of survival outside the ICU. “Patient 2” has a *very high* (80%-100%) chance of survival in the ICU and a *medium* (40%-60%) chance of survival outside the ICU. “Patient 3” has a *high* (60%-80%) chance of survival in the ICU and a *low* (20%-40%) chance of survival outside the ICU. “Patient 4” has a *low* (20%-40%) chance of survival in the ICU and a *very low* (0-20%) chance of survival outside ICU. And “Patient 5” has a *medium* (40%-60%) chance of survival in the ICU and a *very low* (0-20%) chance of survival outside the ICU. As to the level of confidence about the information provided, let us assume the user felt “*confident*” about this.  Figure 2 shows the results obtained by the model considering these input values.

The results in Figure 2 suggest that Patients 2, 5, and 3 should go to the ICU since the robustness index for them is 95%, 93%, and 92%, respectively. This means that in 92% of the simulation instances, Patient 2 was part of the optimal allocation; in 93% of the simulations instances, Patient 5 was in the optimal allocation; and in 92% of the simulation instances, Patient 3 was in the optimal portfolio. The results from the robustness index for Patient 4 and Patient 1 were 10% and 9%, respectively, which means they were part of the optimal portfolio in only 10 and 9% of the simulation instances. In the graphic of Figure 2, green bars mean the patient should go to the ICU, and red bars mean the patient should not go to the ICU. A total of 100,000 scenarios was simulated, which means that 100,000 simulation instances were performed, i.e., in each of these instances, the portfolio selection problem given by equations (6), (7), and (8) was run, and the robustness index of each patient shown in Figure 2 represents the percentage of cases in which that patient belongs to the optimal portfolio, considering all the instances performed.

The values considered for the parameters to obtain the results in Figure 2 were *U*(*S*_IN_) = *U*(*S*_OUT_) = 1, and *U*(*D*_IN_) = *U*(*D*_OUT_) = 0, but these values may be changed whenever necessary. Parameters should be calibrated in conjunction with analysts and/or experts and can be changed in the system.

The results presented here for the ICU allocation problem are for recommendation and decision support purposes, but the final decision is always up to the doctor. The system also asks the user whether or not he/she intends to follow the recommendation provided; but the user is not obliged to answer it. A feedback space is also available for the user when clicking on the “conclude” button.

## 5. Final Remarks

This paper presented a utility-based portfolio selection approach to support doctors in the allocation of intensive care unit beds decision problem, in the light of the complex situation of the absence and lack of resources caused by the COVID-19 pandemic. This problem is very common in health system routines and has a special role in the complex situation brought by the pandemic. The proposed approach has potential impact related to the possibility of tackling this critical decision situation in a rational and structured way, based on proposing and implementing well-founded decision theory techniques for aiding health systems managers. Thus, this model can directly influence in the strategy to save the maximum number of patients, since the rational conduct of these medical decision processes is fundamental to enabling doctors to tackle problems arising from the COVID-19 pandemic scenario.

Moreover, giving attention to these common decision problems prompts consideration also being given to a wide variety of other applications for the proposed approach in this study. The proposed model can continue to be used after the pandemic has been brought under control, this being an important feature constructed in the pandemic period that can help doctors in their routines. It is worth mentioning, however, that the recommendations provided by this system are not normative. In other words, the proposed model is a supplement to support doctors, but it is for doctors to decide whether or not to follow the recommendations provided.

For future research, studies can be performed in order to investigate other mathematical models constructed to deal with this pandemic scenario, including the possibility of considering partial information about preferences [37]. Future works can also investigate the subjective probabilities provided by the doctors in order to perform behavioral studies.

## Figures and Tables

**Table 1:** Previous studies about ICU allocation problem.

Theme	Authors	Contribution
Perceptions and attitudes of health professionals	Giannini and Consonni [18]	Perceptions about inappropriate admissions and resource allocation
	Coomber et al. [19]	Occupational stress of ICU doctors

Techniques or models to support the ICU allocation problem (or similar issues)	Azcarate et al. [23]	Proposition of a framework
	He et al. [20]	Systematic literature review of research design and modeling techniques
	Reiz et al. [21]	Use of big data and machine learning
	Edenharter et al.[17]	Use of logistic regression model
	Cao and Huang [27]	Use of discrete event simulation model
	Shmueli and Sprung [22]	Application about survival benefits of allocating a patient to an ICU
	Almeida et al. [15]	Model to maximize the expected number of lives of patients in a Pediatric ICU

Ethical issues	McGuire and McConnell [25]	Discuss fairness and ethics in the ICU allocation problem
	Ghanbari et al. [29]	Systematic literature review about ethical issues involved in allocating resources
	Oerlemans et al. [24]	Interviews with health professionals concerning ethical problems
	White et al. [26]	Used ethical principles to improve allocation decisions

**Table 2:** Representation of prior probabilities.

	*θ*_1_ = *Patient* *Survives*	*θ*_2_ = *Patient* *does* not *survive*
Patient *i* allocated to ICU	*π*_*i*_(*S*_IN_)	*π*_*i*_(*D*_IN_)
Patient *i* not allocated to ICU	*π*_*i*_(*S*_OUT_)	*π*_*i*_(*D*_OUT_)

## Data Availability

The database generated from the occurrences registered in the Decision System used to support the findings of this study is restricted by the Ethical Committee in Research of the Federal University of Pernambuco with CAAE (“Certificado de Apresentação e Apreciação Ética”-Certificate of Presentation and Ethical Appreciation) number 31065820.5.0000.5208 in order to protect patient privacy.
